# Integromics network meta-analysis on cardiac aging offers robust multi-layer modular signatures and reveals micronome synergism

**DOI:** 10.1186/s12864-015-1256-3

**Published:** 2015-03-04

**Authors:** Konstantina Dimitrakopoulou, Aristidis G Vrahatis, Anastasios Bezerianos

**Affiliations:** Department of Medical Physics, School of Medicine, University of Patras, Patras, 26500 Greece; Department of Computer Engineering and Informatics, University of Patras, Patras, 26500 Greece; Singapore Institute for Neurotechnology (SINAPSE), Center of Life Sciences, National University of Singapore, Singapore, 117456 Singapore

**Keywords:** Cardiac aging, microRNA synergism, Module, Integromics network, Meta-analysis

## Abstract

**Background:**

The avalanche of integromics and panomics approaches shifted the deciphering of aging mechanisms from single molecular entities to communities of them. In this orientation, we explore the cardiac aging mechanisms – risk factor for multiple cardiovascular diseases - by capturing the micronome synergism and detecting longevity signatures in the form of communities (modules).

For this, we developed a meta-analysis scheme that integrates transcriptome expression data from multiple cardiac-specific independent studies in mouse and human along with proteome and micronome interaction data in the form of multiple independent weighted networks. Modularization of each weighted network produced modules, which in turn were further analyzed so as to define consensus modules across datasets that change substantially during lifespan. Also, we established a metric that determines - from the modular perspective - the synergism of microRNA-microRNA interactions as defined by significantly functionally associated targets.

**Results:**

The meta-analysis provided 40 consensus integromics modules across mouse datasets and revealed microRNA relations with substantial collective action during aging. Three modules were reproducible, based on homology, when mapped against human-derived modules. The respective homologs mainly represent NADH dehydrogenases, ATP synthases, cytochrome oxidases, Ras GTPases and ribosomal proteins. Among various observations, we corroborate to the involvement of *miR-34a* (included in consensus modules) as proposed recently; yet we report that has no synergistic effect. Moving forward, we determined its age-related neighborhood in which *HCN3*, a known heart pacemaker channel, was included. Also, *miR-125a-5p/-351, miR-200c/-429, miR-106b/-17, miR-363/-92b, miR-181b/-181d, miR-19a/-19b, let-7d/-7f, miR-18a/-18b, miR-128/-27b* and *miR-106a/-291a-3p* pairs exhibited significant synergy and their association to aging and/or cardiovascular diseases is supported in many cases by a disease database and previous studies. On the contrary, we suggest that *miR-22* has not substantial impact on heart longevity as proposed recently.

**Conclusions:**

We revised several proteins and microRNAs recently implicated in cardiac aging and proposed for the first time modules as signatures. The integromics meta-analysis approach can serve as an efficient subvening signature tool for more-oriented better-designed experiments. It can also promote the combinational multi-target microRNA therapy of age-related cardiovascular diseases along the continuum from prevention to detection, diagnosis, treatment and outcome.

**Electronic supplementary material:**

The online version of this article (doi:10.1186/s12864-015-1256-3) contains supplementary material, which is available to authorized users.

## Background

Aging is an inevitable part of life and unfortunately poses the largest risk factor for cardiovascular diseases. The limited success of medical community in dealing with complex diseases and the increasing population of aging patients with cardiovascular diseases led to rapidly increasing costs of health care that most economies cannot sustain. Advances in personalized medicine and the biology of aging are two important steps towards overcoming the escalating health care costs on the way to a better understanding and treatment of such complex diseases [1]. Cardiac aging is characterized by a continuum of cardiac structural and functional alterations involving left ventricular hypertrophy, diastolic dysfunction, increased risk of atrial fibrillation, valvular degeneration and fibrosis, and decreased maximal exercise capacity. Apparently, the decline in function provoked by the longevity associated changes make the aged heart more susceptible to stress, leading to a high prevalence of cardiovascular diseases and heart failure [2]. As such, in order to extend lifespan, current drug development strategies design anti-aging drugs that delay the implicated age-related diseases or vice versa [3].

In the road for exploring the underlying aging etiology many studies were directed towards discovering age-dependent genes/proteins [4,5]. More recently, other works [6-8] characterized several microRNAs (miRNAs) as center players in cardiac aging process and in the development of multifarious heart diseases, such as heart hypertrophy, arrhythmia, acute myocardial infarction, and heart failure. Recent estimations count around 1100 miRNAs in the human genome that modulate various biological processes ranging from proliferation, differentiation to senescence and apoptosis [9]. Moreover, the small amount of miRNAs is able to regulate a large number of genes through synergism, in which multiple miRNAs work synergistically to regulate individual mRNAs [9-11]. Despite the advances, the underlying mechanisms are still far from known and the next step towards featuring aging is systems-level analyses that will reveal pathways - instead of individual proteins or miRNAs - responsible for transducing stress, mechanical and neurohormonal stimuli into gene expression changes [12].

To this end, approaches from the network perspective capture the complex regulatory mechanisms between mRNAs and miRNAs in a more comprehensive manner. As such, network-based methods to study aging have evolved in parallel with the concept of “omics” and the explosion of high-throughput data. Managbanag *et al.* [13] suggested that proteins linked through shortest paths in the protein interaction network with established age-related genes were more likely to be aging regulators. Budovsky *et al*. [14] showed that hub proteins in the protein-protein interaction network connecting human homologs of lifespan modifiers were significantly linked to age-related diseases. Also, the work of Xue *et al.* [15] depicted the modularity (i.e. existence of dense subnetworks with distinct functional role relative to other subnetworks) of the aging network and contextualized the transcriptional changes during lifespan through a small number of network modules. Moreover, the study of Li *et al.* [9] explored the regulatory effect between miRNAs and mRNAs in the developmental and aging process of the human brain by integrating miRNA and mRNA expression profiles in the form of a bipartite miRNA-mRNA regulatory network and identified modules showing miRNA synergism. We relegate the reader to the review of Hou *et al.* [16] for an extensive view of the network-based methodologies on the study of aging. Nevertheless, the number of network-based studies that deal with the unraveling of mammalian tissue-specific aging mechanisms is small [17-19] and even smaller when the role of microRNA synergism is the question [9].

This integromics network-based meta-analysis study offers the first systemic view of cardiac aging molecular mechanisms and sketches the path for identifying robustly many more other involved molecular components than those already reported in literature. Motivated by recent findings that identified various heart-specific miRNAs as aging modulators, we tested their reproducibility facilitated by the availability of transcriptome, micronome and proteome data in the mouse model. Since a single study often has small sample size and limited statistical power, combining information across multiple studies is an intuitive way to increase sensitivity [17,20-22]. As such, we show that integrating information extracted from multiple independent microarray experiments along with mouse interaction data can produce more accurate cardiac aging signatures in the form of multi-omics subnetworks (modules). Through modularity we revise the role of recently implicated miRNAs and proteins and suggest that their impact on aging is realized via their neighborhoods. In addition, we investigate the miRNA synergism on the modular level and propose miRNAs whose lifespan disturbances generate collaboratively profound biological effects. For this, we developed a synergy score which can be used to assess the nature and scale of miRNA synergy, and considers both the incorporative contribution of miRNA co-regulation on the same biological process (Intra-modular) as well as on different biological processes (Inter-modular). The proposed method offers a theoretical framework and guideline for multi-target combinational miRNA therapy of age-related cardiovascular diseases.

## Results

**Figure 1:**
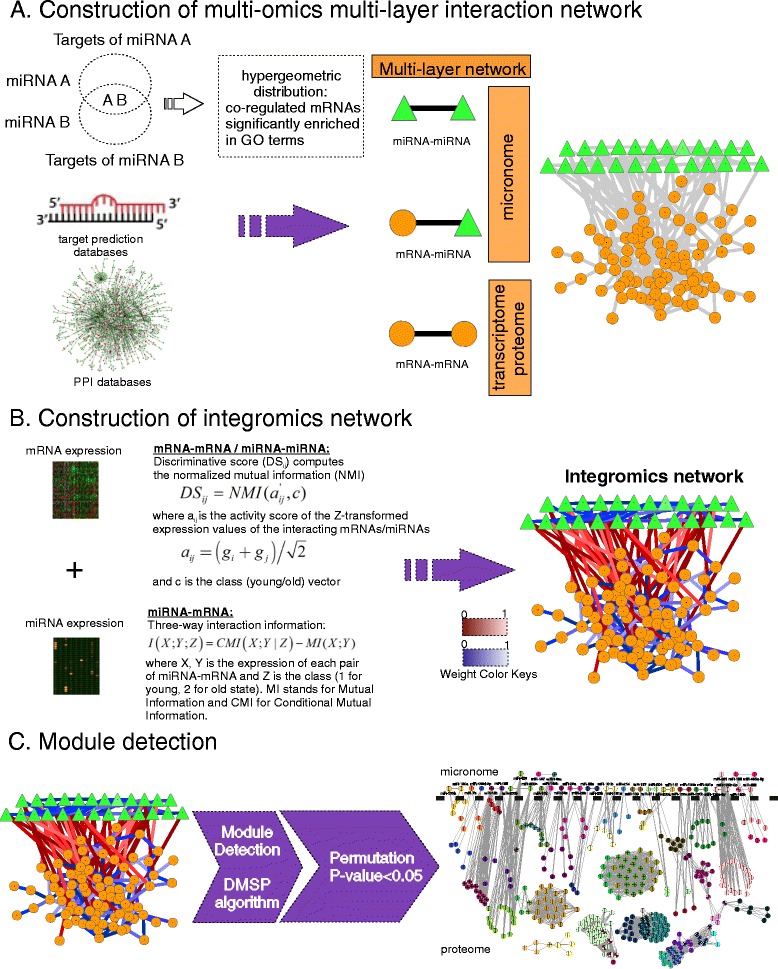
**Methodology workflow for detecting integromics modules.** **A.** Construction of the miRNA-mRNA multi-layer regulatory network based on interaction databases and hypergeometric distribution (in case of miRNA-miRNA relations). **B**. Two adapted weighting schemes were employed to integrate the mRNA/miRNA expression information onto the corresponding network layer so as to create the integromics network. **C**. The integromics network was the input to the DMSP module-detecting algorithm and statistically significant modules (P-value < 0.05) were identified based on a permutation strategy on the expression data.

Our integromics meta-analysis method can be summarized into the following steps: (i) Collection of diverse interaction data (e.g. protein-protein, protein-DNA, miRNA-mRNA) from many databases and prediction tools (in case of miRNA-mRNA relations); (ii) Construction of a multi-layer network with two types of nodes (mRNAs and miRNAs) and three types of relations (mRNA-mRNA, miRNA-mRNA and miRNA-miRNA). We denote that the terms ‘gene’, ‘mRNA’ and their encoded ‘protein’ are used interchangeably in this paper. Also, the hypergeometric distribution was employed to define miRNA-miRNA relations based on significantly functionally associated mRNA targets (Figure 1); (iii) Aggregation of mRNA and miRNA independent cardiac-specific expression datasets from various mouse strains; also, a human mRNA expression experiment was included; (iv) Design of two adapted weighting schemes that integrate the expression information onto the respective interaction network layer; notably, a separate weighted network was constructed for each of the 28 possible combinations of mRNA and miRNA experiments in the mouse model (Figure 1); (v) Application of a module-detecting algorithm on each weighted network that efficiently identified modules substantially altered during lifespan (Figure 1); (vi) Use of meta-analysis method to identify consensus modules across multiple mouse weighted networks and then across organisms; (vii) Evaluation of micronome synergism based on an in-house designed synergy score and employment of Borda voting scheme as meta-analysis method to rank the miRNA pairs.

### Topological analysis of the unweighted multi-layer network

**Figure 2:**
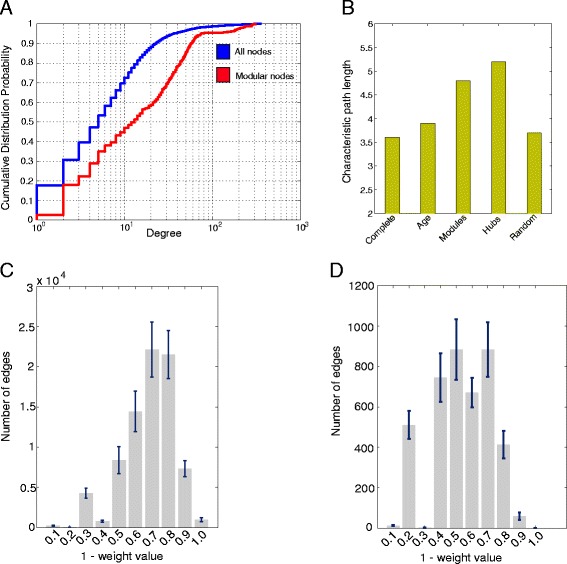
**Degree distribution, weight distribution and topological analysis. A)** Degree distribution of nodes (mRNAs/miRNAs); blue refers to the complete set and red to the fraction of nodes included in the consensus modules. The variance of modular nodes was significantly different (Bartlett test, P-value = 1.4E-39) and with higher mean value, which is translated into more hub nodes favored during module construction. **B)** Calculation of the characteristic path length (CPL) of the complete network, of the network after removing the 186 age-dependent nodes, of the network after removing the 429 consensus modular nodes, of the network after removing the top 429 hub nodes and of the network after removing randomly 429 nodes (mean value after 100 runs). If the nodes removed are important mediators for network communication CPL will increase. **C)** Weight distribution of the complete multi-layer network with bars representing the mean edge weight value as calculated from all combinations of mRNA/miRNA expression experiments and error bars depicting the confidence intervals. Each bar represents a value range named after the upper limit. All weight values were adjusted to ‘1-weight value’ due to the fact that DMSP algorithm constructs modules by promoting edges with weights closer to zero. As shown, with cutoff value ≤ 0.4, only 8% of the complete interactome (~5,300 edges) changed substantially during lifespan **D)** Weight distribution of the edges included in the final consensus modular topology. With cutoff value ≤ 0.4, 1,270 edges were included in the consensus modular topology which represent the 24% of the total age-related relations (one-sided Fisher exact test, P-value = 2.2E-16).

We examined the degree distribution of the unweighted multi-layer network both at each separate layer (mRNA-mRNA, miRNA-mRNA, miRNA-miRNA) as well as whole. We observed that the mRNA-mRNA and miRNA-mRNA layers followed a power law and an exponential distribution (mRNA-mRNA: slope = −1.6, R^2^ (coefficient of determination) = 0.91, miRNA-mRNA: slope = −1.39, R^2^ = 0.83, miRNA-miRNA: slope = −0.7, R^2^ = 0.37). Moreover, the networks displayed scale free characteristics indicating so that there exist core sets of organizing principles in its structure. Moving further, we tested the degree distribution of the nodes participating in the consensus modules relative to the complete set of nodes in the initial network. As observed, the modular nodes exhibited significant variance in degree values (Bartlett test, P-value = 1.4E-39) along with higher mean degree value (Figure 2A). Based on the degree metric we ranked all nodes and, after setting a cutoff at the top 15%, 1,097 mRNAs were defined as hubs and 122 of them were included in the consensus modules. Similarly, 64 miRNAs were characterized as hubs and 18 of them were included in the consensus modules. We denote that mRNAs and miRNAs were treated separately due to the fact that miRNAs exhibit much higher degree values and in other case the degree sorting would be biased in favor of the miRNAs. We observed that the hub nodes (both mRNAs and miRNAs) were over-represented in the consensus modules (one-sided Fisher exact test, P-value = 2.2E-16 and P-value = 8.482E-07 for mRNAs and miRNAs respectively) suggesting so that the majority of independent expression experiments corroborate to the involvement of hub nodes in the longevity mechanisms of cardiac tissue.

To test whether the age-dependent nodes (as estimated by linear regression analysis), consensus modular nodes or hub nodes are important to the multi-layer network stability, we used an established test for network structure stability — recording the changes in characteristic path length (CPL) in the largest connected component of the network after selective node removal (see ‘Methods’ section). If the nodes removed are important mediators for network communication, the route from one node to another within the network will be a longer path and the CPL of the resulting network will increase. This is an important network statistic and represents closeness and consequently how quickly information can be transferred in a network [23]. Targeted removal of nodes according to degree metric is also called ‘attack’ [24]. Attacking hubs in the network, as expected, increases significantly the CPL whereas random removal of nodes from the network maintains the initial CPL values. It is evident from Figure 2B that removal of the consensus modular nodes – nodes involved in modules voted by the majority of coupled mRNA/miRNA expression experiments – affected the stability more than attacking age-dependent nodes (two sample t-test, P-value < 0.01) and less than hub nodes. This finding can be interpreted byway; on one hand, modules have higher discriminative potential than independent differentially expressed age-related nodes to describe the transitions from young to old state, and on second hand, hub nodes participate in the network integrity but in fact only a fraction (those included in the modular topology) is truly responsible for the aging process. In more detail, the consensus modules were significantly enriched in age-dependent proteins (11 out of the 162 characterized as age-dependent) and moreover, our method contextualized them into specific neighborhoods (over-representation was estimated with one-sided Fisher exact test, P-value = 0.01). We note that the consensus modules were not significantly enriched in age-dependent miRNAs.

### Longevity attacks a small fraction of the cardiac interactome

Towards evaluating the scale of longevity effect upon interactome topology, we examined the edge weight distribution of the multiple weighted networks — created based on all possible combinations of mRNA/miRNA expression datasets in the mouse model. After selecting weight value cutoff lower than 0.4, only 8% of the complete interactome (~5,300 edges) changed substantially during lifespan (Figure 2C). This observation disagrees with the results of [25] that showed that environmental stimuli like heat shock disintegrate significant proportion of the yeast interactome and decrease the weight structure. Our suggestion is that gradually developing cumulative processes like aging affect a small proportion of the interactome, which has been proved to be organized into smaller scale highly inter-connected communities. On second level, we zoomed into the edge weight distribution of the 3,780 edges included in the consensus modular topology so as to ensure that consensus modules are both reproducible across independent datasets and at the same time include a significant amount of age-related edges. Interestingly, our method captured, based on the same cutoff value, 1,270 edges which represent the 24% of the total age-related relations (Figure 2D) (over-representation estimated by one-sided Fisher exact test, P-value = 2.2E-16). This finding secures to a great extent that the consensus modules can serve rightfully as signatures of cardiac aging and potential multi-targets for the treatment of age-related heart diseases.

### miRNA synergy in mouse model

**Figure 3:**
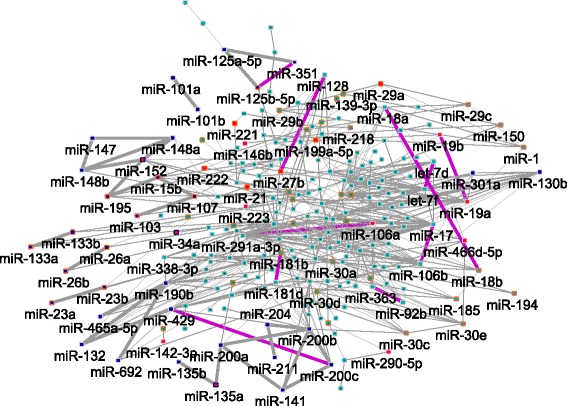
**miRNA-miRNA synergistic network.** This network incorporates the miRNA relations that ranked in the upper quartile (639 edges) of the Borda voting scheme. Blue colored nodes represent miRNAs involved in mouse consensus modules. Edges with larger width highlight the relations included in consensus modules. Red colored nodes represent age-dependent miRNAs as estimated by linear regression analysis. Red colored miRNAs with blue border are both age-dependent and participate in consensus modules. Nodes with orange colored border represent miRNAs associated to human cardiac pathophysiology as reported in miR2Disease database. Purple edges highlight the 10 top scoring miRNA relations.

With the use of an in-house designed metric, we evaluated all miRNA pairs in terms of synergism (MS score) first based on the modular result of each weighted network and second after applying a Borda voting meta-analysis scheme to determine the final rank of each miRNA pair based on all modularized weighted networks (see also Additional file 1). We categorized miRNA pairs into two types: (a) those pairs which ranked in the upper quartile of the complete set of miRNA relations (2,553 relations in total) and participated in the final set of 40 consensus modules and (b) those pairs which ranked in the upper quartile but are not included in the consensus modules. The latter case leaves room for further exploration in advent of more time expression series experiments on cardiac aging and more interaction data, where the specific miRNAs would probably get re-contextualized. In Tables 1 and 2, we report for each type the Borda rank for each pair, the human disease-related information for each miRNA (based on orthologs of miRBase (http://www.mirbase.org)) as well as literature-extracted evidence that relates each miRNA with cardiac pathophysiology or aging [6-8,26-52]. Also, in Figure 3 an overview of the miRNA synergistic network is provided containing all miRNA relations in the upper quartile of the Borda ranking.

**Table 1:** **miRNA synergism results relative to consensus modules**

**miRNA A**	**miRNA B**	**Borda rank**	**Disease A**	**Disease B**	**Literature A**	**Literature B**
miR-125a-5p	miR-351	5	Various cancer types			[26]
miR-200c	miR-429	10	Various cancer types	Various cancer types	[27,28]	[28]
miR-125b-5p	miR-351	112	Cardiac hypertrophy		[29]	[26]
miR-15b	miR-195	174	Cardiac hypertrophy	Cardiac hypertrophy/heart failure	[6]	
miR-148a	**miR-152**	194	Various diseases	Various diseases		[30,31]
miR-200b	miR-200c	199	Various cancer types	Various cancer types	[28]	[28]
miR-132	miR-190b	221	Various diseases		[32]	
miR-190b	miR-465a-5p	261				
miR-190b	miR-692	309				
miR-107	miR-15b	327	Cardiac hypertrophy	Cardiac hypertrophy		[6]
**miR-152**	miR-195	342	Various diseases	Cardiac hypertrophy/heart failure	[30,31]	
miR-148b	**miR-152**	353	Various diseases	Various diseases		[30,31]
miR-132	miR-429	371	Various diseases	Various cancer types		[28]
miR-130b	miR-301a	372	Various cancer types			[33]
**miR-135a**	miR-135b	395	Various cancer types	Various cancer types	[34,35]	
miR-101a	miR-101b	396	Various cancer types			
miR-147	miR-148a	397	Various cancer types	Various diseases	[35]	
miR-125a-5p	miR-125b-5p	400	Various cancer types	Cardiac hypertrophy		[29]
miR-133a	miR-133b	401	Cardiac hypertrophy/cardiomyopathy/myocardial infarction	Myocardial infarction	[36,37]	[36,37]
miR-103	miR-107	407	Cardiac hypertrophy	Cardiac hypertrophy		
miR-141	miR-200a	408	Various cancer types	Various cancer types	[38]	[28]
miR-147	miR-148b	410	Various cancer types	Various diseases		
miR-200a	miR-200b	412	Various cancer types	Various cancer types	[28]	[28]
miR-141	miR-200b	413	Various cancer types	Various cancer types	[38]	[28]
miR-148a	miR-148b	415	Various diseases	Various diseases		
miR-200b	miR-204	427	Various cancer types	Various cancer types	[28]	
miR-141	miR-200c	464	Various cancer types	Various cancer types	[38]	[28]
miR-26a	miR-26b	465	Cardiac hypertrophy	Cardiac hypertrophy		[39]
miR-23a	miR-23b	493	Cardiac hypertrophy	Cardiac hypertrophy		
**miR-135a**	miR-200a	494	Various cancer types	Various cancer types	[34,35]	[28]
miR-204	miR-211	537	Various cancer types	Various cancer types		

miRNA synergism results for the consensus modules according to Borda voting scheme. Each line reports the Borda rank of each miRNA pair identified in the final consensus modules, disease-related information of the human orthologs as recorded in miR2Disease database (if found only cardiac pathophysiology related terms are reported) and evidence related to aging or cardiac pathophysiology provided by recent literature. We denote that in many cases each member of the reported miRNA pair can participate in a different module. **Bold** miRNAs are age-dependent based on linear regression analysis.

**Table 2:** **miRNA synergism results beyond consensus modules**

**miRNA A**	**miRNA B**	**Borda rank**	**Disease A**	**Disease B**	**Literature A**	**Literature B**
*miR-106b*	*miR-17*	1	Various diseases	Various diseases	[40]	[41]
***miR-19a***	***miR-19b***	2	Various cancer types	Various cancer types	[6,42,43]	[6,41-43]
*miR-363*	*miR-92b*	*3*	Alzheimers disease	Lupus nephritis		
miR-181b	miR-181d	4	Cardiac hypertrophy	Various diseases		
*let-7d*	*let-7f*	6	Various cancer types	Various cancer types	[44]	
miR-18a	miR-18b	7	Various cancer types	Cardiac hypertrophy	[42]	
miR-128	**miR-27b**	8	Various diseases	Cardiac hypertrophy		
**miR-106a**	miR-291a-3p	9	Various diseases		[45]	
miR-30a	miR-30e	100	Cardiac hypertrophy	Cardiac hypertrophy	[7,46]	[7]
miR-30e	miR-384-5p	132	Cardiac hypertrophy		[7]	[47]
miR-30c	miR-30e	184	Cardiac hypertrophy	Cardiac hypertrophy	[46]	[7]
miR-294	miR-30e	318		Cardiac hypertrophy		[7]
miR-291a-3p	miR-30e	364		Cardiac hypertrophy		[7]
miR-18b	miR-297c	385	Cardiac hypertrophy			
miR-190	miR-194	391	Various cancer types	Cardiac hypertrophy		[28]
miR-30d	miR-30e	398	Cardiac hypertrophy	Cardiac hypertrophy		[7]
***miR-34a***	*miR-34b-5p*	399	Various diseases	Various cancer types	[8]	
miR-30e	miR-495	411	Cardiac hypertrophy	Various diseases	[7]	[48]
miR-139-3p	miR-150	438	Cardiac hypertrophy	Cardiac hypertrophy		[28]
miR-30e	**miR-466d-5p**	453	Cardiac hypertrophy		[7]	
miR-1	miR-18a	457	Cardiac hypertrophy/cardiomyopathy/heart failure/myocardial infarction/coronary artery disease	Various cancer types	[36]	[42]
miR-187	miR-18b	460	Non-alcoholic fatty liver disease (NAFLD)	Cardiac hypertrophy		
miR-185	miR-18b	462	Cardiac hypertrophy	Cardiac hypertrophy		
miR-100	miR-30e	489	Various diseases	Cardiac hypertrophy	[49]	[7]
miR-1	**miR-19a**	492	Cardiac hypertrophy/cardiomyopathy/heart failure/myocardial infarction/coronary artery disease	Various cancer types	[36]	[6,42,43]
miR-1	miR-206	512	Cardiac hypertrophy/cardiomyopathy/heart failure/myocardial infarction/coronary artery disease	Various diseases	[36]	
miR-194	miR-210	530	Cardiac hypertrophy	Various diseases	[28]	[50]
miR-192	miR-218	548	Various cancer types	Cardiac hypertrophy	[28]	
miR-191	miR-218	549	Various cancer types	Cardiac hypertrophy	[48]	
miR-18b	miR-219	550	Cardiac hypertrophy	Various cancer types		
miR-18b	miR-22	552	Cardiac hypertrophy	Various diseases		[26,51]
***miR-34a***	*miR-449b*	563	Various diseases	Various cancer types	[8]	
miR-150	miR-224	570	Cardiac hypertrophy	Various cancer types	[28]	
miR-18b	miR-297b-5p	599	Cardiac hypertrophy			
miR-150	miR-296-5p	620	Cardiac hypertrophy	Various diseases	[28]	
miR-1	**miR-29a**	634	Cardiac hypertrophy/cardiomyopathy/heart failure/myocardial infarction/coronary artery disease	Cardiac hypertrophy	[36]	[52]
miR-1	miR-29b	636	Cardiac hypertrophy/cardiomyopathy/heart failure/myocardial infarction/coronary artery disease	Cardiac hypertrophy	[36]	[6,52]
miR-1	miR-29c	639	Cardiac hypertrophy/cardiomyopathy/heart failure/myocardial infarction/coronary artery disease	Cardiac hypertrophy	[36]	[6,52]

Synergism results for miRNA pairs that ranked in the upper quartile according to Borda voting scheme but not incorporated in the consensus modules (indicative examples). Each line reports the rank of each miRNA pair, disease-related information of the human orthologs as recorded in miR2Disease database (if found only cardiac pathophysiology related terms are reported) and evidence related to aging or cardiac pathophysiology provided by recent literature. **Bold** miRNAs are age-dependent based on linear regression analysis. We also added miRNA pairs with high rank yet not related to aging or cardiac pathophysiology (highlighted in *italics*).

We note that 47 miRNAs associated to human cardiac diseases, according to miR2Disease database, were found in the upper quartile of the Borda rank and 11 of them also involved in the 40 consensus modules. We initially zoom onto *miR-34a* motivated by the study of Boon *et al.* [8] which named it as cardiac aging biomarker (the dataset of the study is used in our analysis). Our results support the involvement of this miRNA in cardiac aging since it was both characterized as age-dependent and included in consensus modules; nevertheless its synergism effect was not substantial (relations including *miR-34a* were over 399^th^ rank). Further, *miR-22* was suggested as cardiac aging biomarker by the work of Jazbutyte *et al.* [26]. Our analysis failed to identify this miRNA in the consensus modules and its synergism impact is not substantial (>551^th^ rank). The explanation behind this observation lies both in the experimental settings of the original study as well as in the later fold-change-based method to detect differentially expressed molecules. On one hand, when the original miRNA expression dataset was combined with other mRNA expression datasets *miR-22* was radically repositioned relative to its targets and their expression profiles. Moreover, the fold-change method by definition excludes molecules with moderate differential expression yet important players in longevity mechanisms. Second, standard statistical methods to define differentially expressed molecules fail to incorporate dependencies among molecules and more importantly how the dependencies – instead of single entities – change during aging.

Nevertheless, the same study identified *miR-351* as age-dependent but - as stated by the authors - it was not chosen for validation experiments. On the contrary, our results indicate that the synergistic effect of this miRNA and its partner *miR-125a-5p* is much higher (5^th^ rank). Also, we comment on the *miR-200c/-429* pair (10^th^ rank). The works of Sataranatarajan *et al.* [27] and Schraml and Grillari [28] suggested their involvement in kidney aging but to our knowledge no association to cardiac tissue aging has been reported.

Interesting are the observations regarding the miRNA-miRNA edges that failed to be incorporated in the consensus modules yet their synergism was substantial based on Borda meta-analysis results. We denote that synergy results are meant to be evaluated separately from the consensus modular results based on the different scopes and on the different meta-analytic schemes employed. On one hand, the consensus set was defined after majority voting among modularized networks while meta-analysis on synergy aimed at pinpointing edges with substantial macroscopic effect on the topology across datasets but without considering their involvement into reproducible modules. The reason is that there were many cases where specific miRNA edges affected multiple pairs of modules which in turn were not always sufficiently reproducible, as pairs, across datasets, i.e. involved in the consensus set. However, we account for the synergistic effect of these edges since the majority of expression experiments agree on their substantial impact. We comment on the *miR-106b/-17* pair which acquired the first rank. Age-related evidence for *miR-106b* is found in the work of Brett *et al.* [40] which associated it with neural stem cell proliferation and differentiation during aging. With respect to *miR-17* we relegate the reader to the review of Mogilyansky and Rigoutsos [41] which links this miRNA to a variety of diseases such as cardiovascular and aging. The same study validates the presence of *miR-19b* in the *miR-19a/-19b* pair which ranked second and both miRNAs were defined as age-dependent. Moreover, the work of van Almen *et al.* [42] linked *miR-19a* and *miR-19b* with age-related heart failure. Further, we comment on *miR-27b* (*miR-128/-27b* pair appeared on the 8^th^ rank) which was shown to be up-regulated to different degrees in the old versus young adult heart and was induced during early hypertrophic growth in response to pressure-overload [6]. Similar synergism hypotheses can be derived for the rest of miRNA pairs reported in Tables 1 and 2, which are supported in terms of aging or cardiac pathophysiology by recent literature evidence.

### Consensus mouse modular signatures

**Figure 4:**
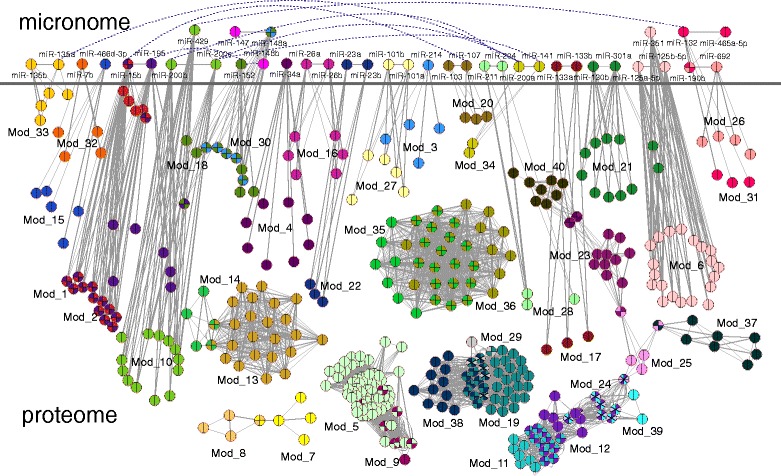
**Mouse consensus modules.** Layout of the 40 consensus modules. The topology includes 3,780 edges among 38 miRNAs and 391 proteins. The multi-layer modules are visualized in two levels, i.e. micronome and proteome. Nodes in many cases are multi-colored (with the use of ExprEssence Cytoscape plug-in by Warsow *et al.* [53]) in a pie-chart-like form so as to visualize the participation of a node in multiple modules. Blue dotted edges highlight miRNA relations not included into consensus modules yet exhibited significant synergy.

In order to produce highly reproducible modular signatures, we constructed and modularized multiple weighted networks based on all possible combinations of mRNA/miRNA expression experiments so as to reduce the effect of different mouse strains, platform arrays, laboratory effects and gender. The consensus set included 40 modules (see Additional file 2) identified in at least 18 out of the 28 combinations (Figure 4 [53]). The consensus modular topology contained 38 miRNAs and 391 mRNAs. We checked the genes for statistically significant Gene Ontology (GO) terms with DAVID functional tool [54] and the enriched biological process terms were: *‘generation of precursor metabolites and energy’* (EASE score, modified Fisher exact P-value = 3.0E-32), *‘electron transport chain’* (P-value =1.4E-26), *‘cell cycle phase’* (P-value = 2.1E-12) and *‘organelle fission’* (P-value = 4.0E-12). The respective GO terms highlight processes commonly reported in cardiac aging studies [55,56]; they also provide feedback that the integromics meta-analysis approach is efficient in zooming out and providing the broader view around genes/proteins well-associated to cardiac aging - as individual molecular components - but not relative to their interactors.

When genes were grouped into modules only 8 modules were significantly enriched (P-value ≤ 0.05) with GO biological process terms (Table 3). Also, 6 longevity-associated genes according to GenAge database were identified in the consensus modules: *BUB1B* (budding uninhibited by benzimidazoles 1 homolog, beta (*S. cerevisiae*)), *ERCC2* (excision repair cross-complementing rodent repair deficiency, complementation group 2), *SLC25A4* (solute carrier family 25), *UCP2* (uncoupling protein 2), *MCM2* (minichromosome maintenance deficient 2 mitotin (*S. cerevisiae*)) and *EPS8* (epidermal growth factor receptor pathway substrate 8).

**Table 3:** **Analysis of mouse consensus modules**

**Module index**	**Count**	**Gene ontology term**	**Percentage**	**Fisher exact P-value**	**Age-dependent miRNAs**	**Age-dependent genes**	**GenAge genes**
1	18	One-carbon metabolic process	11.8	5.0E-2	N/A	N/A	N/A
2	22	Cellular aromatic compound metabolic process	10	5.0E-2	N/A	GOS2	
3	5	N/A	N/A	N/A		N/A	N/A
4	9	N/A	N/A	N/A	miR-34a	N/A	N/A
5	68	Generation of precursor metabolites and energy	73.5	1.6E-77	N/A	HADH, NDUFA10, NDUFV1	SLC25A4, UCP2
6	23	N/A	N/A	N/A	N/A	CDH22	N/A
7	4	N/A	N/A	N/A	N/A	N/A	N/A
8	5	Heart development	40	5.0E-2	N/A	N/A	N/A
9	8	N/A	N/A	N/A	N/A	NDUFA10	N/A
10	16	Tissue development	30.8	7.2E-3	N/A	N/A	EPS8
11	20	N/A	N/A	N/A	N/A	N/A	BUB1B
12	27	N/A	N/A	N/A	N/A	N/A	BUB1B, MCM2
13	24	N/A	N/A	N/A	N/A	RHOU	N/A
14	5	N/A	N/A	N/A	N/A	N/A	N/A
15	6	N/A	N/A	N/A	miR-466d-3p	IER3	N/A
16	8	N/A	N/A	N/A	N/A	N/A	N/A
17	5	N/A	N/A	N/A	N/A	N/A	N/A
18	13	N/A	N/A	N/A	miR-152	N/A	N/A
19	36	N/A	N/A	N/A	N/A	RPL10, RPL37	N/A
20	5	N/A	N/A	N/A	N/A	N/A	N/A
21	13	N/A	N/A	N/A	N/A	N/A	N/A
22	5	N/A	N/A	N/A	N/A	N/A	N/A
23	13	Response to DNA damage stimulus	53.8	1.4E-8	N/A	N/A	ERCC2
24	8	N/A	N/A	N/A	N/A	N/A	MCM2
25	6	N/A	N/A	N/A	N/A	RPA3	N/A
26	6	N/A	N/A	N/A	N/A	N/A	N/A
27	7	N/A	N/A	N/A	N/A	N/A	N/A
28	5	N/A	N/A	N/A	N/A	N/A	N/A
29	7	N/A	N/A	N/A	N/A	EEF2	N/A
30	8	N/A	N/A	N/A	N/A	N/A	N/A
31	6	N/A	N/A	N/A	N/A	N/A	N/A
32	5	N/A	N/A	N/A	N/A	N/A	N/A
33	6	N/A	N/A	N/A	miR-135a	N/A	N/A
34	4	N/A	N/A	N/A	N/A	N/A	N/A
35	28	Cellular nitrogen compound metabolic process	78.6	6.2E-12	N/A	N/A	N/A
36	25	Cellular nitrogen compound metabolic process	76	2.1E-9	N/A	N/A	N/A
37	8	N/A	N/A	N/A	N/A	N/A	N/A
38	24	N/A	N/A	N/A	N/A	N/A	N/A
39	10	N/A	N/A	N/A	N/A	N/A	MCM2
40	10	N/A	N/A	N/A	N/A	N/A	N/A

Summary of the 40 consensus modules. Count: The number of module members; Gene Ontology (GO) term: Statistically significant GO terms based on DAVID tool (P-value <= 0.05); Percentage: The percentage of modular genes characterized by the specific GO term; Fisher exact P-value: modified Fisher exact P-value (EASE score) as provided by DAVID (the complete mouse genome was used as background); Age-dependent miRNAs: Age-dependent miRNAs based on linear regression analysis; Age-dependent genes: Age-dependent genes based on linear regression analysis; GeneAge genes: longevity-associated genes according to GenAge database. N/A: not available.

### Human cardiac aging modular signatures

**Figure 5:**
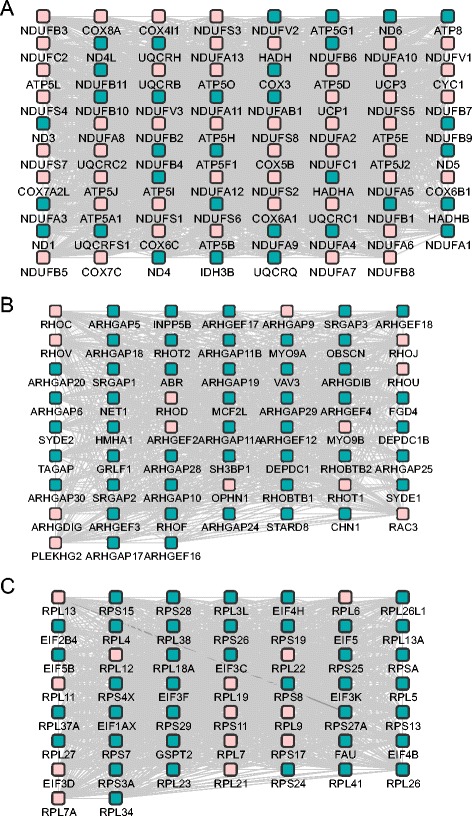
**Homology-based human modules.** Layout of the three human modules descending after the homology-based matching between human and mouse consensus modules. Module **A** displayed significant node overlap relative to mouse Mod_5, module **B** to Mod_13 and module **C** to Mod_19. Pink nodes highlight the homologs.

The mouse consensus modules were mapped with the use of NCBI's Gene and HomoloGene resources against the human modules [57] and the node overlap was estimated only with respect to genes due to the lack of human miRNA expression experiments. Nevertheless, the overlap threshold (NOR) was lowered to 0.3 due to the absence of miRNAs and the insufficiency of homologs. The matching procedure revealed three human modules that matched the Mod_5, Mod_13 and Mod_19 (Figure 5). The first human module matched to Mod_5 was characterized by the *‘electron transport chain’* GO term (EASE score, modified Fisher exact P-value <0.05) similar to the mouse corresponding one. The second human module was significantly enriched in the *‘signal transduction’*, *‘actin cytoskeleton organization’* and *‘programmed cell death’* (P-value <0.05) terms relative to Mod_13 where no statistically significant terms were found. The third human module was enriched in the *‘protein metabolic process’* term relative to the mouse Mod_19 where no statistically significant terms were found. Also, we checked the 189 human modular proteins for heart disease terms and 13 proteins were found to be associated (Table 4) (over-representation estimated by one-sided Fisher exact test, P-value = 0.05).

**Table 4:** **Disease annotation data for proteins included in human modules**

**Gene**	**Gene name**	**Disease name**
NDUFS2	NADH dehydrogenase Fe-S protein 2	Cardiomyopathies
NDUFV2	NADH dehydrogenase flavoprotein 2	Cardiomyopathies
ARHGAP9	Rho GTPase activating protein 9	Coronary Vasospasm
HADHB	hydroxyacyl-CoA dehydrogenase	Heart Diseases
ATP5J	ATP synthase, H+ transporting, mitochondrial Fo complex, subunit F6	Heart Diseases
ARHGDIB	Rho GDP dissociation inhibitor (GDI) beta	Acute Coronary Syndrome
RHOJ	ras homolog family member J	Cardiomyopathies
NDUFS1	NADH dehydrogenase Fe-S protein 1	Cardiomyopathy, Hypertrophic
UCP3	uncoupling protein 3	Heart Failure/Hypertrophy, Right Ventricular
ATP5I	ATP synthase, H+ transporting, mitochondrial Fo complex, subunit E	Heart Diseases
COX5B	cytochrome c oxidase subunit Vb	Myocardial Ischemia/Cardiomegaly
NDUFA2	NADH dehydrogenase 1 alpha subcomplex, 2	Coronary Disease
NDUFB3	NADH dehydrogenase 1 beta subcomplex, 3	Coronary Disease

Cardiovascular disease annotation data for 13 proteins participating in the human modules. Data was obtained from DisGeNET database.

### Age-related mRNAs/miRNAs

Although the goal of this study is to detect age-related modules, we employed conventional statistical tests for identifying age-dependent mRNAs/miRNAs so as to explore later their place into modules. Using a linear regression model (see ‘Methods’ section), 24 miRNAs and 162 mRNAs were identified as age-dependent (F-test, P-value < 0.05), whose expression level was significantly altered with age. Gene Ontology (GO) biological process enrichment analysis with the use of DAVID functional tool revealed the following terms (EASE score, modified Fisher exact P-value < 0.05) for 89 out of the 162 genes: *‘positive regulation of defense response’*, *‘regulation of growth’*, *‘regulation of developmental process’*, *‘regulation of binding’* and ‘*regulation of response to stress’*. We comment on the *‘stress’* and *‘defense’* related terms which characterize the rate of aging and the appearance of age-related pathology which are modulated by stress response and repair pathways that gradually decline, including the proteostasis and DNA damage repair networks and mitochondrial respiratory metabolism [58].

In Table 5 we report the age-dependent mRNAs/miRNAs as well as highlight the 11 (out of 162) mRNAs and 4 (out of 24) miRNAs that were identified in the final consensus modules. The age-dependent miRNAs, included in the consensus modules, have been reported to be related to aging or specifically to cardiac aging based on recent studies: *miR-34a* [8], *miR-466d-3p* [6], *miR-152* [30] and *miR-135a* [34]. Similar literature evidence support 10 age-dependent genes/proteins included in consensus modules: *G0S2* (G0/G1 switch gene 2) has been related to cardiac hypertrophy [59]; *HADH* (hydroxyacyl-CoA dehydrogenase) involved in fatty acid oxidation is associated to cardiac hypertrophy [60]; *NDUFA10* (NADH dehydrogenase 1 alpha subcomplex 10) and *NDUFV1* (NADH dehydrogenase flavoprotein 1) are involved in oxidative phosphorylation and associated to cardiovascular diseases [61]; *RHOU* (ras homolog gene family, member U) is responsible for changes in cytoskeleton and cell shape during aging [62]; *IER3* (immediate early response 3) is involved in heart failure [63]; RPL10 (ribosomal protein L10) and *RPL37* (ribosomal protein L37) reflect the decline in ribosomal activity in the aging heart [64]); *RPA3* (replication protein A3) mediates critical DNA transactions throughout the genome [65]; *EEF2* (eukaryotic translation elongation factor 2) is involved in the slowing down of protein synthesis during cardiac aging [66].

**Table 5:** **Age-dependent mRNAs/miRNAs**

**List of age-dependent miRNAs F-test (P-value < 0.05)**	**List of age-dependent genes F-test (P-value < 0.05)**
miR-106a	1810046J19RIK, 2310044G17RIK, 2900073G15RIK, ACOT1, AKR1C13,
**miR-135a**	ALAS1, ALDOB, AMPD3, AMY1, ANKRD1, ANXA5, AOX1, APBB1, BAIAP2L1, BAX, BLCAP, C1QA, C3, CAT, CCND1, CCNDBP1, CDC37, CDC37L1, **CDH22,** CLU, CNOT8, COL5A2, CORIN, CP, CRHR2, CSDA, CTGF, CTSD, CXCL14, D0H4S114, D930014E17RIK, DAP, DAP3, DAZAP2, DCI, DECR1, DNAJA1, DNAJC5, DYNLL1, EDN3, **EEF2,** EFHD2, EIF4EBP1, FAH, FBP2, FGL2, FIGF, FXYD6, **G0S2,** GABARAPL1, GLUL, GP49A, GPR56, GRINA, GSTA4, GUCY1B3, H19, **HADH,** HDLBP, HIF1A, HIST1H1C, HIST1H2BC, HNRPDL, HOMER2, HSPA8, HSPH1, HTATIP2, HTRA1, **IER3,** IRAK1, ITGB6, ITM2B, IVNS1ABP, KCNK1, KLF9, LAMP1, LGALS4, LGMN, LRRC3B, LYZ2, MID1IP1, MLF1, MLYCD, MORF4L1, MRPL45, MRPL50, MXD4, MYADM, MYOM2, MYOT, **NDUFA10, NDUFV1,** NEDD1, NFKBIA, NNT, NT5E, OSMR, PAH, PCDHB20, PEG3, PER1, PFDN1, PGAM1, PIM3, POPDC3, POSTN, PPAP2B, PRKCQ, PRNP, PROX1, PSMB4, PTER, PTP4A3, PTTG1IP, RAB4A, RCAN1, RELA, REXO2, RFXANK, **RHOU, RPA3, RPL10,** RPL23, **RPL37,** SCARB2, SCN1B, SDPR, SEC63, SEPP1, SERPINA3N, SERPINH1, SH3GL2, SLC35A2, SLC3A2, SLC4A8, SMAD4, SNX3, SOCS3, SQSTM1, STAG2, STAP2, STAT5A, TCEA1, TCTA, TEF, TFRC, TGM2, TMSB4X, TNFAIP8, TPP1, TRPM7, TSPAN17, TSPAN4, UBA3, UNG, XPR1, YWHAE
miR-142-3p	
miR-146b	
**miR-152**	
miR-199a-5p	
miR-19a	
miR-19b	
miR-21	
miR-221	
miR-222	
miR-223	
miR-27b	
miR-290-5p	
miR-29a	
*miR-342-3p*	
**miR-34a**	
***miR-466d-3p***	
*miR-374*	
*miR-466c-5p*	
*miR-466f-3p*	
*miR-468*	
*miR-574-5p*	
*miR-677*	

Using a linear regression model and two-tailed F-test, 24 age-dependent miRNAs and 162 age-dependent mRNAs were identified (P-value < 0.05). **Bold** indicates miRNAs and mRNAs included in the consensus modules. *Italics* indicate miRNAs with insignificant synergistic effect.

Our suggestion is that age-dependent molecules, as derived by classic statistical tests and/or supported by one independent study, are of little value on their own. Alternatively, age-dependent molecules gain value within the boundaries of modules that as a whole change significantly during lifespan even if their neighbors display moderate differential expression and thus have a lower discriminative potential. Under this notion, the modular approach filtered the differentially expressed molecules, as derived from classic statistical tests, to a smaller more reliable set and more importantly contextualized them into neighborhoods. From the synergism perspective, we deescalated the value of several miRNAs descending from classical differential expression analysis; we showed that differential expression is not synonymous or indicative of synergism.

## Discussion

Accumulating evidence shows the broad impact of miRNAs in modulating complex physiological or disease phenotypes. A cohort of recent studies has stressed out the pervasive role of miRNAs in the analysis of cardiovascular diseases and shifted the interest to miRNAs as rational drug targets and, as such, miRNA-based therapy represents an essential and promising trend in the future [67]. Moreover, aging is the predominant risk factor for cardiovascular diseases and contributes to a significantly worse outcome in patients with acute myocardial infarction [68]. In this orientation, this work is motivated by recent findings revealing novel cardiac aging biomarkers at the level of individual proteins and miRNAs in the mouse model [6,8,26]. However, there is still no meta-analysis study to assess the validity and reproducibility of these observations and even less is known about the functional association and interactions among molecular components. We address this challenge and integrate cardiac tissue miRNA and mRNA expression profiles from multiple independent studies with various interaction data and constructed multiple integromics networks that served as the basis for defining consensus communities (modules) as cardiac aging signatures. Unlike conventional clustering or classification methods, network-based methods can implicate proteins with low discriminative potential (e.g., those that are not differentially expressed) if such proteins participate in a community whose overall activity is discriminative [69]. Such proteins can arise within a significant community if they are essential for maintaining its integrity, that is they are required to interconnect many hub proteins.

An important aspect of our network analysis is the construction of the miRNA-miRNA interaction network and the identification of extensive synergies that affect profoundly the aging process and decline of cardiac tissue. Previous studies have inserted the term ‘synergy’, in the context of miRNA regulation, to describe pairs of miRNAs that significantly co-regulate at least one functional module [70]. Moving a step forward, we developed an integrated parameter synergy score which can be used to assess the complex nature and scale of miRNA synergy in the genome. This unique scoring system measures the contribution of a miRNA pair both to a specific process (Intra-modular) and to multiple processes (Inter-modular). Initially, we re-address and complement to the findings of recent studies that identified miRNAs involved in heart tissue aging process. The work of Boon *et al.* [8] suggested that *miR-34a* inhibition reduces cell death and fibrosis following acute myocardial infarction and improves recovery of myocardial function. We support the longevity-associated role of *miR-34a* since it participated in the consensus modules, yet showed no substantial involvement in synergism. The associated modular genes were: *CUEDC1* (CUE domain containing 1), *FCGR2B* (Fc receptor), *ACCN1* (amiloride-sensitive cation channel 1), *HCN3* (hyperpolarization-activated cyclic nucleotide-gated K + 3), *PADI2* (peptidyl arginine deiminase type II), *4930453N24RIK* (predicted gene), *PNOC* (prepronociceptin) and *P2RY2* (purinergic receptor G-protein coupled 2). *HCN3* has been suggested to be involved in cardiac aging and decline since it is the pacemaker and modulates activity in the contractile myocytes [71]. Also, the relation of *miR-34a* with *PNOC* has been reported in brain aging as proposed by Somel *et al.* [72].

Moving forward, *miR-22* was shown to be involved in age-related cardiac fibrosis, whose overexpression contributed to cellular senescence and migration of cardiac fibroblasts [26]. Our findings do not add value to its involvement in aging process (not present in consensus modules) and its synergy impact was not substantial (*miR-18b/-22* pair appeared on the 552^th^ rank). Nevertheless, *miR-351* proposed as aging biomarker by the same study - but not further experimentally validated – was shown to have significant synergy effect on aging process (*miR-125b-5p/-351* pair appeared on the 112^th^ rank) and was incorporated in consensus modules. The module around *miR-351* included, among others, proteins such as *CBS* (cystathionine β-synthase), *MMP11* (matrix metalloproteinase 11) and *NEU1* (lysosomal sialidase 1). Damage on *CBS* has been shown to lead to decreased H2S production and concentration during aging [73]. The work of [74] has suggested that the differential regulation of *MMPs* is associated with aging and hypertension in the rat heart. *NEU1* has been proposed to participate in the elastin degradation during vascular aging and provoke atherosclerosis [75].

Another previous study showed that the members of *miR-17-92* cluster, including *miR-18a*, −*19a*, and *-19b*, were differentially expressed in failure-prone heart of aged mice as well as in cardiac biopsies of idiopathic cardiomyopathy patients at old age with severely impaired cardiac function [42]. We corroborate to this observation since the *miR-19a/-19b* and *miR-18a/-18b* pairs scored high in terms of synergy (2^nd^ and 7^th^ rank respectively) despite not participating in the consensus modules. In summary, the miRNA synergy analysis provided a list of new candidate miRNA pairs both included and not included in the consensus modules. After reviewing the literature we found evidence that link most of the top-scoring miRNA pairs (until 10^th^ rank) to aging and cardiovascular diseases and consider them reliable for further validation.

Most importantly, our analysis revealed a modularized view of the aging process in cardiac tissue and established the ground for a more holistic perspective of the complex regulatory processes taking place in cardiac tissue during lifespan. Initially, we comment on the scale of longevity effect upon the interactome. Our results showed that only a small fraction of the network edges is affected during lifespan in contrast to other studies that evaluated the effect of heat shock on yeast interactome and observed global disintegration [25]. However, we argue that such global changes are probably prominent in cases when the biological system is severely under attack like in heat shock; aging is a gradual cumulative process and should probably be evaluated on a smaller scale and through perturbations on specific areas of the interactome.

Moving forward, the set of 40 consensus modules descended from various independent mRNA and miRNA expression studies along with the applied weighting schemes increased the homogeneity of the module compositions and ensured a high probability of identifying members with both correlating and anti-correlating profiles during age transition from young to old; thus, the derived observations can be regarded as more confident and realistic. The topological analysis suggests that modules affect more profoundly the stability of the network compared to individual age-dependent nodes and less when compared to hub nodes. This observation stresses out an important advantage of module-based analysis into describing aging process; it allows the resulting communities to display mixed features like (non)hubness, include proteins placed in the periphery - which in other cases would be neglected - and contain both differentially expressed nodes and insignificantly differentially expressed nodes which however affect longevity. The GO enrichment analysis of modules elucidated well-established cardiac age-related terms like: *‘generation of precursor metabolites and energy’*, *‘electron transport chain’*, *‘cell cycle phase’* and *‘organelle fission’*. We relegate the reader to the work of Houtkooper *et al.* [76] which exemplifies that metabolic dysfunction is a common hallmark of aging.

Moving further, considering that modules regulating aging in model systems may not be related to human aging, mouse consensus modules were matched against human based on homologs. The comparison revealed three mouse consensus modules displaying significant overlap with the respective human. Gene Ontology analysis on the corresponding human modules elucidated terms like *‘electron transport chain’*, *‘signal transduction’*, *‘actin cytoskeleton organization’*, *‘programmed cell death’* and *‘protein metabolic process’*. We comment on the term *‘actin cytoskeleton organization’* which has been suggested to be linked to downstream signaling events that further modulate cellular activity, and which can determine cell fate, the regulation of programmed cell death, the maintenance of homeostasis and the process of cellular aging [77,78]. In yeast, for example, it has been shown that the level of damage sustained by the actin cytoskeleton under oxidative stress is directly related to apoptosis. Further evidence descends from observations that actin-based propulsion mechanisms are required for the inheritance of mitochondria and anti-ageing factors into newly formed cells. In addition, actin is known to directly influence the formation of protein aggregations [77]. Moving forward, the proteins participating in these modules are mainly NADH dehydrogenases involved in the respiratory chain, mitochondrial ATP synthases involved in oxidative phosphorylation, cytochrome oxidases, Ras superfamily GTPases and ribosomal proteins. NADH dehydrogenases have been suggested to associate with heart aging [79], ATP synthases are linked with the changes in ATP supply in advanced age [80], decrease of cytochrome oxidases mRNA transcripts during aging has been reported in the rat heart [81], the association of Ras superfamily with longevity has been implicated in the work of Borras *et al.* [82] while declining ribosomal activity may be a feature of aging in the heart [64]. Further, we explored the hypothesis that genes/proteins related to cardiac pathophysiology do not necessarily influence the aging process in cardiac tissue. Our results suggest that this is partially true since 13 out of the 189 proteins included in the human modules are related to various cardiac diseases. At this point we relegate the reader to the review of North and Sinclair [83] which summarizes how the genetic pathways that regulate aging in model organisms influence the cardiovascular health state.

There are some limitations in our study. First, the platform differences at probe-level among experiments and the genome-scale coverage reduced significantly the intersecting genes among experiments and thus the size of the protein interaction graph under study. In this sense, many other consensus modules could appear if the protein network expanded in size. Also, the small number of publicly available mouse and human expression experiments recording the age phases of cardiac tissue affects the impact of our results. More value will be added to our methodological framework in advent of more comprehensive data. Despite these limitations, our study still provides a new insight into the synergism of miRNAs in cardiac aging and offers a more reliable pool of multi-layer aging signatures for further experimental validation.

## Conclusions

This works offers the first systemic view of cardiac aging mechanisms well known to be highly interconnected with many cardiovascular diseases. For this, we propose a meta-analysis network-based methodology that integrates proteome and micronome interaction data along with transcriptome expression data from multiple independent studies to produce robust modular signatures of longevity mechanisms, in contrast to the individual molecular components proposed by each transcriptome study separately. Moving a step forward, we explore the micronome synergism from the modularized network perspective and propose miRNA pairs with profound collective action during cardiac aging. The meta-analysis findings revise the role of several recently implicated molecules and re-contextualize them into communities with high reproducibility across datasets and organisms. The proposed methodological framework can be applied in a wide range of complex cellular processes and diseases, and can subvene combinational multi-target miRNA therapy of age-related cardiovascular diseases.

## Methods

### Datasets

#### Interaction datasets

All human and mouse biomolecular interactions were downloaded with the use of MiMI Cytoscape plug-in [84] where we selected all molecule types and all data sources. Also, protein interactions were collected with the use of iRefR package in Bioconductor [85]; for each organism (mouse or human) we isolated the interactions where both nodes belonged to the same reference organism. A universe of miRNA-mRNA interactions for all reported mouse miRNAs was constructed as a composite of all validated interactions downloaded from TarBase (http://diana.cslab.ece.ntua.gr/tarbase) and predicted targets reported by miRecords (http://miRecords.biolead.org). The predicted component integrates the predictions produced by 11 established miRNA target prediction programs (DIANA-microT, MicroInspector, miRanda, MirTarget2, miTarget, NBmiRTar, PicTar, PITA, RNA22, RNAhybrid, and TargetScan⁄TargertScanS). Predictions were filtered to only consider those targets predicted by at least 4 of 11 prediction algorithms (as proposed in the works of [86,87]). The TarBase validated interactions were then added to the filtered list and duplicated interactions were eliminated. Information about the human miRNA-disease network was obtained from miR2Disease database (http://www.mir2disease.org/), human protein disease data was obtained from DisGeNET database (http://www.disgenet.org/) and list of mouse age-related proteins was downloaded from GenAge database (http://genomics.senescence.info/genes/).

#### Expression datasets

mRNA and miRNA expression datasets were obtained from the Gene Expression Omnibus database (http://www.ncbi.nlm.nih.gov/geo/) and from published studies. The analyzed data represent a comprehensive collection of most experiments (9 in total) that have evaluated the effect of aging in the laboratory mouse or human samples, including the cardiac-specific samples generated as part of the AGEMAP project [88] (see Additional file 3). To our knowledge, there is no human public miRNA expression dataset that records the age effect in cardiac tissue and for this the miRNA synergism was not explored in this case. Only age-related data from healthy, non-treated specimens was analyzed and data from specific diseases, treatments and mutants were excluded. We denote that heart age-specific experiments exist also in other organisms like rat model; however, the lack of sufficient biomolecular interactions hampers the comparison among organisms and therefore the rat model was excluded from analysis.

Most data were generated using Affymetrix oligonucleotide microarray platforms, but Agilent, miRCURY and non-commercial arrays were also used in some experiments. All experiments were normalized with global scaling, MAS 5.0, Z-transformation, lowess or quantile method except the dataset of Park et al. [89] which was quantile normalized. If more than 30% of measurements for a given probe contained nulls or missing values, the probe was excluded. Otherwise, null values were replaced by the probe’s average (row average method) and probes targeting the same gene were averaged. In case an experiment contained male and female samples, the dataset was split and each subset was treated independently throughout analysis, in order to ensure that the module detecting procedure is not affected by gender factor. All datasets, except those already processed, were log2 and Z-transformed. Also, we checked in each dataset for failed samples; for this, we created an all-by-all matrix of how well each sample’s expression profile correlates with the profiles of the remaining samples in the same age group and excluded samples whose average Pearson’s correlation was lower than 0.7. While these differences in pre-processing were not ideal, the deleterious effect of such differences and batch effects was minimized by analyzing all experiments independently, without combining expression scores from different studies.

### Multi-layer unweighted network

#### Construction of multi-layer network

With the term ‘multi-layer network’ we define a graph including two types of nodes (mRNAs and miRNAs) and three types of relations (mRNA-mRNA, miRNA-mRNA and miRNA-miRNA). We denote that the terms ‘gene’, ‘mRNA’ and their encoded ‘proteins’ are used interchangeably in this paper. The miRNA-miRNA layer was constructed based on the hypothesis that their co-regulating targets are highly enriched in the same Gene Ontology (GO) biological process terms [90]. The GO file on Biological Processes was downloaded from the GO consortium (http://geneontology.org/) and biological process categories were restricted to below the fourth level of the hierarchy to avoid analyzing very general non-descriptive terms. In detail, for a given miRNA pair A and B, we identified the target subset they co-regulate *A*_*target*_ ∩ *B*_*target*_. The over-represented biological processes of the target subset are defined according to hypergeometric cumulative distribution. The probability *PG*_*i*_ for *A*_*target*_ ∩ *B*_*target*_ in the GO term *i* is calculated as:

1 $$ {\mathrm{PG}}_{\mathrm{i}}=1-F\left(x\Big|N,{K}_i,M\right)=1-{\displaystyle \sum_{t=0}^x}\frac{\left(\begin{array}{c}\hfill {K}_i\hfill \\ {}\hfill t\hfill \end{array}\right)\left(\begin{array}{c}\hfill N-{K}_i\hfill \\ {}\hfill M-t\hfill \end{array}\right)}{\left(\begin{array}{c}\hfill N\hfill \\ {}\hfill M\hfill \end{array}\right)},\kern0.5em i=1,2,\dots, I $$

where *N* is the number of all targets (default background distribution), *K*_*i*_ is the total number of genes that are annotated in the GO term *i* and targeted by miRNAs, *M* is the size of *A*_*target*_ ∩ *B*_*target*_, *x* is the number of targets in *A*_*target*_ ∩ *B*_*target*_ that are also annotated to term *i* and *I* is the total number of GO terms. The PG score was computed for each miRNA pair and for each available GO term characterizing the intersecting target mRNAs. The significant miRNA synergistic pairs were defined after setting PG ≤ 0.05 for at least 9 GO terms.

The mRNA-mRNA layer was formed after examining the expression datasets for intersections in terms of Entrez Ids. After checking all combinations we selected the intersection of the GSE11291, GSE43556 and Park et al. [89] datasets which contained 10,084 common IDs. For this gene subset, the mRNA-mRNA level included 14,560 interactions among 3,396 nodes after removing self-loops. The miRNA-mRNA layer included 45,802 relations among 421 miRNAs and 6,195 mRNAs while the miRNA-miRNA layer contained 2,553 interactions among 396 miRNAs. After removing smaller components, the final graph included 62,915 interactions among 7,321 nodes (see Additional file 4). We note that for the remaining expression datasets (i.e. outside the intersection) a separate multi-layer network was constructed which was in all cases smaller in terms of nodes and edges; however, we state that the 2,553 miRNA-miRNA interactions were present in all network cases.

For the analysis of the human transcriptome dataset the network was constructed only with regard to proteins and the final topology contained 176,700 interactions among 16,336 nodes (see Additional file 4).

#### Topological analysis of the multi-layer unweighted network

The topological characteristics of the unweighted network were analyzed with the NetworkAnalyzer Cytoscape plug-in [91]. The degree of a mRNA (or miRNA) is the number of its total connections in the layer under investigation. Node degree distribution P(k) is defined as the number of nodes with a degree k for k = 0, 1, 2,… NetworkAnalyzer considers data points with positive coordinate values for fitting the line where the power law curve of the form y = βx^a^. The R^2^ value is a statistical measure of the linearity of the curve fit and used to quantify the fit to the power line. When the fit is good, the R^2^ is close to 1.

Also, we tested the stability of the unweighted network after attacking nodes of interest and recorded the changes on characteristic path length (CPL). CPL is defined as the average of the shortest path lengths between any two nodes:

2 $$ CPL=\frac{2}{N}{\displaystyle \sum_{i\in N}\frac{{\displaystyle {\sum}_{j\in N\ j\ne i}{d}_{ij}}}{N-1}} $$

where *N* is the number of all nodes, *d*_*ij*_ the shortest path length between *i* and *j*, defined as the minimum number of links traversed to get from node *i* to node *j*.

### Multi-layer integromics weighted network

The proposed integromics network approach combines the transcriptome, proteome and micronome information in the form of a composite graph, upon which a module-detecting algorithm will define multi-layer modules with two types of nodes (mRNAs and miRNAs) and three types of relations (*mRNA-mRNA, miRNA-mRNA, miRNA-miRNA*). For each type of relation we applied an adapted weighting scheme based on the notion that the aim is to identify the putatively age-related communities that change substantially in the transition from young to old state. We denote that for the mouse model, there were 28 possible combinations of mRNA/miRNA expression data to be laid as information onto the interactome network and consequently 28 weighted networks were produced.

*mRNA-mRNA/miRNA-miRNA:* An adapted metric was employed that promotes the interacting nodes that co-express (co-activate or co-repress) and simultaneously discriminate significantly in terms of expression between the two states (young/old). In detail, we assigned a non-negative weight to each interaction, which descends after processing the expression profiles of the corresponding molecules. For each mRNA or miRNA, the expression profiles in both classes (young/old) were reshaped in a vector followed by another vector representing the corresponding class (*c*) labels. Next, the activity score (*α*) of each interacting pair of mRNAs or miRNAs *i* and *j* was computed as:

3 $$ {a}_{ij}=\frac{g_i+{g}_j}{\sqrt{2}} $$

where *g*_*i*_ and g_j_ are the Z-transformed expression values. The activity score is discretized (*a’*_*ij*_) into ⌊log 2(# *of samples*) + 1⌋ equally spaced bins as described in [69,90]. The adapted discriminative score (*DS*_*ij*_) computes the normalized mutual information (*NMI*) between *a*’ and *c*:

4 $$ D{S}_{ij}=NMI\left({a}_{ij}^{\hbox{'}},c\right) $$

A reason for using an information-theoretic metric is to deal with the limited number of samples available in aging expression dataset [92] and the reason for using specifically NMI is to capture the potential heterogeneity of expression in age samples, that is, differences not only in the mean but in the variance of expression (available Matlab codes at http://biosignal.med.upatras.gr). The *DS* ranges in [0,1] with larger values indicating strong correlation and at the same time substantial change from young to old.

*miRNA-mRNA:* Based on accumulated evidence that a miRNA may positively correlate with its target mRNA, we used a metric that considers both the correlation and anti-correlation patterns between mRNA and miRNA nodes and the simultaneous differentiation from young to old state. For three variables X, Y, Z, the three-way interaction information I is defined as [93]:

5 $$ I\left(X;Y;Z\right)=CMI\left(X;Y\Big|Z\right)-MI\left(X;Y\right) $$

where X, Y is the expression value of each member of the miRNA-mRNA pair and Z is the class (1 for young, 2 for old state). MI stands for Mutual Information and CMI for Conditional Mutual Information.

#### Modularization on the multi-layer integromics weighted network

Each weighted network was partitioned into overlapping modules with the use of Detect Module from Seed Protein (DMSP) algorithm [94] after adjusting all weights to ‘1-weight’ (DMSP considers weights closer to zero during module construction). The basic operation of this algorithm is to identify a module on the weighted graph by expanding a kernel node set, which originates from a given ‘seed’ node used as starting-point. In our case, all mRNA and miRNA nodes served as seeds. The parameters of DMSP were set after repetitive trials as p_1_ = 0.5 and p_2_ = 0.6 to avoid over-sized modules (>80 members). For permutation testing, we randomly shuffled expression values for all mRNAs/miRNAs 1000 times and calculated the average weight of modules in random conditions. For each module, the significant P-value was the percentage of cases where the average weight was lower than the value in the real condition (P-value < 0.05).

### miRNA synergy score

**Figure 6:**
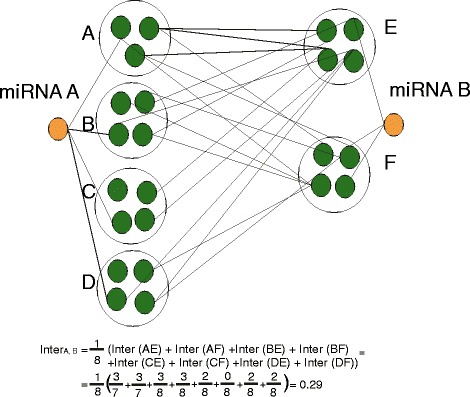
**Estimation of** ***Inter*** **miRNA synergy.** Example illustration for calculating *Inter* miRNA synergy for miRNA pairs not included in the same module. Suppose miRNA **A** targets modules **A**, **B**, **C**, **D** and miRNA **B** targets modules **E** and **F**. There are 8 possible pairs AE, AF, BE, BF, CE, CF, DE and DF to be examined for shared edges between modules and the number of union proteins. In this example the Inter score was estimated to 0.29.

Accumulated evidence suggests that despite their limited number, miRNAs are responsible for evolutionarily robust regulatory effects through coordinated collective actions. As such, attacking a miRNA that is part of a broader functional group will not be as detrimental as assigning each miRNA a unique task. In this orientation, we explored the miRNA synergism from the modular perspective. We supposed that miRNA synergy can be dissected into two types: (a) the intra-modular regulatory effect of a miRNA pair upon the same biological process, i.e. participating in the same module and (b) the inter-modular regulatory effect of a miRNA pair upon multiple biological processes, i.e. each member of the pair is involved in a different module. The following formula is used to calculate MS score for any given miRNA pair miRNA *i* and miRNA *j*:

6 $$ M{S}_{ij}={n}_1\cdot Intr{a}_{ij}+{n}_2\cdot Inte{r}_{ij} $$

where *Intra is* calculated after dividing the number of modules containing a given miRNA pair with the total number of identified modules in the graph. As *Inter*, we define:

7 $$ Inte{r}_{ij}=\frac{1}{k}{\displaystyle \sum_{p=1}^k}\frac{Nshared\kern0.2em \mathrm{interactions}\kern0.2em \mathrm{between}\kern0.2em {p}_i\mathrm{and}\kern0.2em {p}_j}{Nproteins\; of\;{p}_i{\displaystyle \cup }{p}_j} $$

where *k* denotes all possible combinations of module pairs in which *miRNA*_*i*_ and *miRNA*_*j*_ participate. *p*_*i*_ and *p*_*j*_ represent the modules of one possible pair regulated by *miRNA*_*i*_ and *miRNA*_*j*_ respectively. In order to more clearly illustrate the calculation process of *Inter*, a graphical example presentation is provided (Figure 6). In case *Inter* did not range in [0,1], we scaled the results in that range. *n*_*1*_ and *n*_*2*_ are used as parameters for the contribution of *Inter* and *Intra* to the *MS* score. In this study we selected after repetitive trials *n*_*1*_ = 0.6 and *n*_*2*_ = 0.4 to promote the regulatory impact of intra-modular miRNA synergism.

### Meta-analysis of modularized weighted networks

The comparison of modules across datasets and organisms was realized based on the node overlap ratio, defined as [95]:

8 $$ NOR\left({M}_i,{M}_j\right)=2\cdot \frac{M_i{\displaystyle \cap }{M}_j}{M_i+{M}_j} $$

where *M*_*i*_ and *M*_*j*_ represent the compared modules derived from two modularized networks respectively. We note that analysis on overlapping edges was not conducted due to the fact that the module-detecting algorithm by definition preserves, during the implementations across datasets, the same interactions among a specific subset of nodes (see [94] for more details).

After filtering procedure, only 40 modules displayed NOR ≥ 0.7 in at least 18 out of the 28 modularized weighted networks. We checked the frequency distribution of consensus modules to ensure that no biases were introduced by specific combinations of experiments and more specifically by the Park et al. dataset which generated the majority of the combinations. At this point we denote that the majority of consensus nodes were present both in the initial large network (GSE11291, GSE43556 and Park et al. [89]) as well as in the smaller scale networks (GSE8146, GSE75 and GSE9902). The consensus module was defined as the smallest module (in terms of nodes) among the overlapping modules derived by the combinations. With respect to human dataset, the final human modules were defined after looking for modules with high overlap between both male and female dataset. Also, NCBI’s Gene (http://www.ncbi.nlm.nih.gov/gene/) and HomoloGene (http://www.ncbi.nlm.nih.gov/homologene) resources were employed to check the degree of overlap among mouse consensus and human modules only with respect to proteins [57]. We denote that the overlap threshold was lowered to 0.3 due to the absence of miRNAs and the insufficiency of common nodes (homologs).

Regarding synergy scores, the MS values were calculated separately for each modularized weighted network and then the Borda count voting scheme [96] was applied to rank the miRNA-miRNA relations. The Borda count is one of the most famous and intuitive rank aggregation schemes used frequently in bioinformatics meta-analysis methods [97,98]. In detail, each element in each ordered list is given a score depending on its rank and then these weights are summed up across all such lists. Elements in the aggregated list are given in descending order according to the overall scores.

### Age-dependent mRNAs/miRNAs

Beyond the scope of this study, that is to find age-related modules, we employed classical statistical tests for identifying age-dependent mRNAs/miRNAs so as to explore later their place into modules. For each dataset, we first tested the hypothesis that the expression of a given mRNA/miRNA is associated with age. We denote that with respect to Park et al. [89] experiment the analysis was conducted separately for each strain and age-dependent genes were set if supported by at least three strains. Independent analysis was also conducted in case male and female specimens are available and putative genes were considered only if were characterized as statistically significant in both genders.

Similar to the study of [57] we performed a linear regression for each mRNA/miRNA using the equation:

9 $$ {Y}_{ij}={\beta}_{0j}+{\beta}_{1j}Ag{e}_i+{\varepsilon}_{ij} $$

where *Y*_*ij*_ is the signal intensity of mRNA (or miRNA) *j* in sample *i*, Age_*i*_ is the age of the sample *i*, and ɛ_*ij*_ is the error term. The coefficients β_0_ and β_1_ were estimated by least squares. The derived differential expression was evaluated in terms of statistical significance with a two-tailed *F*-test to determine whether the slope of the curve is different than zero, which would indicate a link between the expression signal and age. Genes supported by at least three experiments and miRNAs supported by all experiments along with a *P*-value below 0.05 were characterized as putatively age-dependent.

## Additional files

Additional file 1:
**Borda count ranking results of the 2,553 miRNA pairs based on the synergy scores as evaluated from 28 weighted networks in the mouse model.** In bold the miRNA pairs identified in the consensus modules are highlighted. Additional file 2:
**Consensus modular interactions.** Every interaction is accompanied by the respective module index. Duplicate interactions may exist in case an interaction is present in multiple modules. Additional file 3:
**Summary of the microarray datasets used in the study.**
Additional file 4:
**Mouse and human interactome data.**
